# Continuous surveillance of drug-resistant TB burden in Rwanda: a retrospective cross-sectional study

**DOI:** 10.1093/inthealth/ihac039

**Published:** 2022-06-02

**Authors:** Yves Habimana-Mucyo, Augustin Dushime, Patrick Migambi, Innocent Habiyambere, Jean Claude Semuto Ngabonziza, Tom Decroo

**Affiliations:** Tuberculosis and Other Respiratory Communicable Diseases Division; HIV/AIDS, Disease Prevention and Control Department; Rwanda Biomedical Centre, Kigali, Rwanda; Tuberculosis and Other Respiratory Communicable Diseases Division; HIV/AIDS, Disease Prevention and Control Department; Rwanda Biomedical Centre, Kigali, Rwanda; Tuberculosis and Other Respiratory Communicable Diseases Division; HIV/AIDS, Disease Prevention and Control Department; Rwanda Biomedical Centre, Kigali, Rwanda; Tuberculosis and Other Respiratory Communicable Diseases Division; HIV/AIDS, Disease Prevention and Control Department; Rwanda Biomedical Centre, Kigali, Rwanda; National Reference Laboratory, Department of Biomedical Services, Rwanda Biomedical Centre, Kigali, Rwanda; School of Medicine and Pharmacy, Department of Clinical Biology, College of Medicine and Health Sciences, University of Rwanda, Kigali, Rwanda; Unit of HIV and TB, Department of Clinical Sciences, Institute of Tropical Medicine Antwerp, Antwerp, Belgium

**Keywords:** prevalence, rifampicin-resistant TB, surveillance, surveys, Xpert MTB/RIF

## Abstract

**Background:**

Since the roll-out of the Xpert MTB/RIF assay, continuous surveillance can provide an estimate of rifampicin-resistant TB (RR-TB) prevalence, provided high drug susceptibility testing (DST) coverage is achieved. We use national data from Rwanda to describe rifampicin DST coverage, estimate the prevalence of RR-TB and assess its predictors.

**Methods:**

Routinely collected DST data were entered into an electronic TB case-based surveillance system. DST coverage was calculated among all bacteriologically confirmed pulmonary TB patients notified from 1 July 2019 to 30 June 2020 in Rwanda. The prevalence of RR-TB was estimated among those with DST results. Univariable and multivariable analysis was performed to explore predictors for RR TB.

**Results:**

Among 4066 patients with bacteriologically confirmed pulmonary TB, rifampicin DST coverage was 95.6% (4066/4251). RR-TB was diagnosed in 73 patients. The prevalence of RR-TB was 1.4% (53/3659; 95% CI 1.09 to 1.89%) and 4.9% (20/406; 95% CI 3.03 to 7.51%) in new and previously treated TB cases, respectively. Predictors of RR-TB were: (1) living in Kigali City (adjusted OR [aOR] 1.65, 95% CI 1.03 to 2.65); (2) previous TB treatment (aOR 3.64, 95% CI 2.14 to 6.19); and (3) close contact with a known RR-TB patient (aOR 11.37, 95% CI 4.19 to 30.82).

**Conclusions:**

High rifampicin DST coverage for routine reporting allowed Rwanda to estimate the RR-TB prevalence among new and previously treated patients.

## Introduction

The WHO recommends high TB burden countries to regularly (ideally every 5 y) conduct a TB drug resistance survey (DRS) or use continuous surveillance relying on routinely collected drug susceptibility testing (DST) data to estimate the prevalence of drug-resistant TB (DR-TB).^1^ Periodic surveys may lead to selection bias by only sampling a subgroup and may miss localized outbreaks. Moreover, results from periodic surveys show very large CIs.^1^ Nevertheless, surveys are useful in settings where continuous surveillance is not yet effectively implemented and can provide detailed sociodemographic information and show resistance patterns that are sometimes not identified through routine surveillance. In addition, these surveys could be used to evaluate the laboratory capacity, sample transportation system, data management system and the reliability of routinely collected information. However, the logistics and required budget of the implementation of periodic surveys are a burden to most low- and middle-income settings. As a result, many countries lack updated prevalence estimates from surveys.^2^ In cases where continuous surveillance is used, at least rifampicin resistance (RR) testing should be effectively implemented and cover at least 80% of pulmonary bacteriologically confirmed TB cases. The roll-out of Xpert MTB/RIF testing at decentralized level is a great opportunity to achieve high testing coverage. The WHO also recommends systematic DST for isoniazid and second-line drugs when RR is diagnosed.^1^ To date, only a few countries have used this approach on a national scale. Eritrea showed that a high testing coverage is feasible in participating health facilities; however, 14 of 77 health facilities did not participate.^3^ In Zimbabwe, only a quarter of all notified patients were tested with molecular DST.^4^ Also, in Cameroon, <80% of bacteriologically confirmed cases were included, even although the study period only comprised 6 wk.^5^

In Rwanda, during the last 25 y, because of logistic and financial barriers, only three DRSs were conducted. The estimated prevalence of RR-TB among new TB patients increased from 1.3% (95% CI 0.7 to 2.1%) in 1993 to 3.9% (2.5 to 5.7%) in 2005, then decreased to 1.4% (0.7 to 2.1%) in 2015, with a persistently high prevalence among previously treated TB patients (few previously treated patients were tested in 1993, 9.4% [3.2 to 15.6%] in 2005 and 10.7% [5.0 to 19.4%] in 2015).^6–8^ Since 2015, the country has not been able to repeat the DRS due to financial limitations. Therefore, the National Tuberculosis Programme (NTP) decided to estimate the DR-TB burden using its routinely collected DST data, with its main focus on RR-TB. Through a network of 68 Xpert MTB/RIF machines, covering the territory of the whole country, rifampicin DST is performed systematically in new and previously treated cases. Among those with RR-TB, resistance to fluoroquinolone and second-line injectables is tested to estimate the frequency of pre-XDR and XDR-TB.^9^ The implementation of an individual patient electronic TB database, locally known as the TB case-based surveillance system, embedded within the District Health Information Software 2 (DHIS2) platform, enables the Rwandan NTP to follow-up the collected DST data. This system has been operational in all health facilities since July 2019 but has never been evaluated. Here, we evaluate DR-TB continuous surveillance among newly diagnosed and previously treated cases with bacteriologically confirmed pulmonary TB in Rwanda. We describe DST coverage among eligible TB cases, estimate the prevalence of RR-TB and assess predictors of RR-TB in Rwanda. Among those with RR-TB, we report results for second-line DST.

## Methods

This is a retrospective cross-sectional study using routinely collected TB data from the Rwanda TB case-based surveillance system.

Our study included all TB patients notified countrywide during one fiscal year in the TB case-based surveillance system. Table 1 shows the tests available, and the level of care at which they are available. To achieve a high coverage of rifampicin DST, Xpert MTB/RIF testing was decentralized to all district hospitals. The largest district hospitals had more than one Xpert platform. Fresh sputum samples were transported between peripheral facilities and district hospitals, and from district hospitals to the National Reference Laboratory (NRL) when RR was detected on Xpert. Xpert MTB/RIF was the first test in high-risk groups and in all patients with presumptive TB in Kigali City. Active case finding was in place for contacts of patients diagnosed with TB and prisoners. A system of performance-based financing using key indicators, such as rifampicin DST coverage, motivated the staff. Quarterly TB data verification and validation identified health facilities with poor performance. Regular supervision visits and on-the-job training were organized to support them.

**Table 1.** Tests for the diagnosis of TB and resistance: level of implementation and indications

	Level	Current indications
Smear microscopy	All 202 CDTs in Rwanda.	Any TB presumptive other than HRG and Kigali City.
Xpert MTB/RIF (Classis or Ultra)	Xpert testing sites (68 sites including 52 hospitals). All district hospitals had at least one Xpert testing platform to cover all CDTs in its catchment area.	• Xpert MTB/RIF was used as the initial diagnostic test for high-risk groups (presumptive TB among people living with HIV, TB contacts, prisoners, children aged <15 y and older people aged >55 y) in Rwanda and all patients with presumptive TB in Kigali City. • Follow-up test in all patients diagnosed with TB on smear microscopy and who were not eligible to have Xpert MTB/RIF as initial test, for the diagnosis of RR-TB.
First-line LPA	National Reference Laboratory.	Any patients diagnosed with RR-TB.
Second-line LPA		
Phenotypic DST		

Abbreviations: CDT, centre for TB diagnostic and treatment; DST, drug susceptibility testing; HRG, high risk groups; LPA, line-probe assay; RR-TB, rifampicin-resistant TB.

All pulmonary TB patients with bacteriological confirmation registered from 1 July 2019 to 30 June 2020 were considered as the denominator for the estimation of rifampicin DST coverage. Only those patients with a confirmation of mycobacterium TB (MTB) detection during rifampicin DST were considered for the analysis of rifampicin DST results. Those with RR-TB on either Xpert MTB/RIF, first-line line-probe assay (LPA) or phenotypic DST were referred for isoniazid DST and second-line drug DST (fluoroquinolone, second-line injectables and ethionamide).

The source of information for our study was the routinely collected TB data through the DHIS2 platform. These data are regularly collected and entered in the electronic individual records system, a TB case-based surveillance system, by health facility staff. The NTP organizes TB data verification and validation sessions on a quarterly basis at health facility level by crosschecking data in the system with source data (patient files and registers, laboratory results forms, etc.) and uses the same database for its periodic (quarterly and annual) reporting.

Starting from the routinely used database, a study database was constituted. Variables retrieved included gender, age, type of profession, comorbidities (HIV, diabetes), TB contact history, TB treatment history, method of TB confirmation (bacteriologically confirmed/clinically diagnosed), site of disease (pulmonary/extra-pulmonary) and results for Xpert MTB/RIF, first- and second-line LPA, culture and phenotypic DST. The study database did not include the names or addresses or any other identifiers.

We used Stata 13.0 software (STATA Corp., College Station, TX, USA) for statistical analysis. To calculate the DST coverage, all pulmonary TB patients with bacteriological confirmation registered from 1 July 2019 to 30 June 2020 served as the denominator and those effectively tested as the numerator. To estimate the prevalence of resistance to specified TB drugs, the number of those with resistance to a specific drug or combination of drugs was divided by the total number of those patients with confirmation of MTB detection and with available DST results for a specific drug or combination of drugs. In case of discordance between methods, resistance overrode susceptibility. Cohen's kappa was used to calculate the observed agreement between DST methods. A 95% CI was calculated around the estimate of DR-TB prevalence. The two-sample test of proportions was used to assess whether the estimate for the RR-TB prevalence differed from the one obtained with the 2015 survey. Risk factors for RR-TB (among all those with a RMP DST result) were estimated using multivariable logistic regression. First, an univariable logistic regression analysis was performed. Second, only factors associated with having RR-TB in univariable analysis (p<0.05) were included in a multivariable model. Variables with p<0.05 in the multivariable model were retained as factors significantly associated with having RR-TB.

## Results

**Figure 1.**
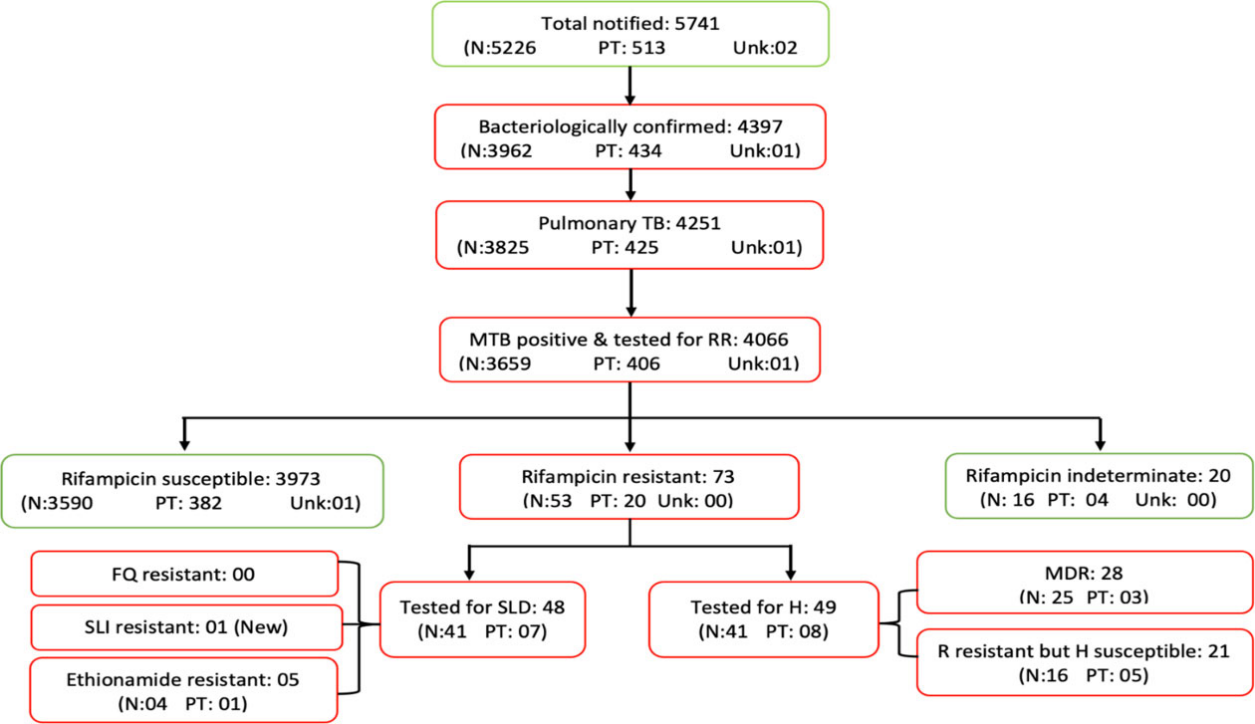
Flowchart of enrolled patients with drug susceptibility testing coverage and results. H, isoniazid; MDR, multidrug-resistant; N, new; PT, previously treated; R, rifampicin; SLD, second-line drugs; Unk, unknown history.

In Rwanda, 5741 TB cases were notified in the study period (July 2019 to June 2020) and 4066 had bacteriologically confirmed pulmonary TB. Rifampicin DST coverage was 95.6% (4066/4251), with 95.7% (3659/3825) and 95.5% (406/425) coverage among new and previously treated cases, respectively (Figure 1).

RR-TB was diagnosed in 73 patients, of whom 71 were diagnosed with Xpert. One additional patient was diagnosed via LPA and another with phenotypic DST (Table 2).

**Table 2.** Concordance between rifampicin drug susceptibility testing results from Xpert, line probe assay and phenotypic testing

	LPA	LPA		
Rifampicin	RS	RR	LPA not done	Agreement (%)
Xpert RS (3959)	0	0	3959	4/6=66.7%
Xpert RR (71)	2	4	65	
Xpert RI (20)	0	0	20	
Xpert not done (16)	12	1	3	
Rifampicin	pDST	pDST	pDST not done	Agreement (%)
	RS	RR		
Xpert RS (3959)	0	0	3959	26/41=63.4%
Xpert RR (71)	15	26	30	
Xpert RI (20)	0	0	20	
Xpert not done (16)	2	1	13	

Abbreviations: pDST, phenotypic drug susceptibility testing RI, rifampicin indeterminate; RR, rifampicin resistant; RS, rifampicin susceptible.

Of 41 (57.7% of 71 with RR on Xpert) with both Xpert and phenotypic DST results, RR-TB was confirmed in 26 (63.4% of 41).

The prevalence of RR-TB was 1.4% (53/3659; 95% CI 1.09 to 1.89%) and 4.9% (20/406; 95% CI 3.03 to 7.51%), respectively, in new and previously treated TB cases (Table 3). There was a significant reduction of RR-TB prevalence from 10.7% in 2015 to 4.9% in 2020 (p=0.04) among previously treated cases. There was no significant difference among new TB cases, 1.5% in 2015 vs 1.4% in 2020 (p=0.81) (Table 4).

**Table 3.** Prevalence of rifampicin-resistant TB

TB treatment history^${\$}$^	Total, n (%)	RS-TB, n (%)	RR-TB, n (%)	Prevalence of RR-TB^#^ (%)	95% CI
New	3659 (90)	3606 (90.3)	53 (72.6)	1.45	1.09 to 1.89%
Previously treated	406 (10)	386 (9.7)	20 (27.4)	4.93	3.03 to 7.51%

^${\$}$ ^Unknown for one patient diagnosed with rifampicin-susceptible TB.

^#^Percantage with 95% confidence interval.

**Table 4.** Comparison of rifampicin-resistant TB prevalence identified during the 2015 survey and 2020 surveillance

Period	Total cases	Prevalence of RR-TB (%)	95% CI	p-value^${\$}$^
New cases				
2015	1033	1.50	0.76 to 2.24	0.81
2020	3659	1.45	1.02 to 1.78	
Previously treated				
2015	84	10.70	4.09 to 17.31	0.040
2020	406	4.93	2.80 to 7.00	

^${\$}$ ^Two-sample test of proportions.

In the multivariable model, three factors were associated with having RR-TB: (1) living in Kigali City, (2) previous TB treatment and (3) close contact with a known RR-TB patient increased the odds of RR-TB by twofold, fourfold and 11-fold, respectively (Table 5).

**Table 5.** Predictors of having rifampicin-resistant TB

	Total	RR-TB					
	n	n (%)	OR	95% CI	aOR	95% CI	p value*
**All**	4066	73 (1.8)	73 (1.8)	4066			
Gender						NS	NA
Female	1129	18 (1.6)	Ref				
Male	2937	55 (1.9)	1.18	0.69 to 2.01			
**Age, y**						NS	NA
0–34	1777	34(1.9)	Ref				
≥35	2289	39(1.7)	0.89	0.56 to 1.41			
**BMI^${\$}$^, Kg/m^2^**						NS	NA
No underweight (≥18)	2524	53 (2.1)	Ref				
Underweight (<18)	1541	20 (1.3)	0.61	0.37 to 1.03			
**HIV status**						NS	NA
Negative	3233	52 (1.6)	Ref				
Positive	833	21 (2.5)	1.58	0.95 to 2.64			
**Contact of RR-TB^${\$}$^**							
No	4032	68 (1.7)	Ref				
Yes	33	5 (15.2)	10.41	3.9 to 27.77	11.37	4.19 to 30.82	<0.001
**TB treatment history^${\$}$^**							
New	3659	53 (1.4)	Ref				
Previously treated	406	20 (4.9)	3.53	2.09 to 5.96	3.64	2.14 to 6.19	<0.001
**Contact of TPB+**						NS	NA
No	3636	63 (1.3)	Ref				
Yes	430	10 (2.3)	1.35	0.69 to 2.65			
**Diabetic**						NS	NA
No	2514	42 (1.7)	Ref				
Yes	26	1 (3.8)	2.35	0.31 to 17.78			
Unknown			1.18	0.74 to 1.89			
**Mineworker^${\$}$^**						NS	NA
No	4016	72 (1.8)	Ref				
Yes	49	1 (2.0)	1.14	0.16 to 8.38			
**Patient location**							
Living in Kigali	1288	31 (2.4)	1.61	1.01 to 2.57	1.65	1.03 to 2.65	0.037
Living outside of Kigali	2778	42 (1.5)	Ref				

Abbreviations: BMI, body mass index; NA, not applicable; NS, not significant; RR-TB, rifampicin-resistant TB; TPB+, bacteriologically confirmed pulmonary TB cases.

*Chi-squared test.

^${\$}$ ^Missing data for one patient for each of those variables.

None out of 28 community health workers or 23 health facility workers had RR-TB (data not shown in Table 5).

Figure 1 shows that, of 73 patients with RR-TB, 49 had results for isoniazid DST and 28 (57.1%) had MDR-TB (resistance to both rifampicin and isoniazid). Second-line DST results were available for 48 (65.8%) RR-TB cases. The agreement between Xpert MTB-XDR and phenotypic DST was 91.9% for isoniazid and 97.3% for both fluoroquinolones and kanamycin (Table 6). No fluoroquinolone resistance was detected, while one patient had RR-TB plus resistance to all second-line injectable agents, and five patients had RR-TB resistant to ethionamide.

**Table 6.** Agreement between drug susceptibility results on Xpert XDR and phenotypic drug susceptibility testing

	phenotypic DST			
	Susceptible	Resistant	Not done	Observed agreement (%)^${\$}$^
**Isoniazid**				
Xpert HS (15)	10	0	5	34/37=91.9%
Xpert HR (27)	2	24	1	
Xpert HI (01)	1	0	0	
Xpert not done (30)	2	3	25	
**Fluoroquinolone^#^**				
Xpert FQS (41)	36	0	5	36/37=97.3%
Xpert FQR (00)	0	0	0	
Xpert FQI(02)	1	0	1	
Xpert not done (30)	5	0	25	
**Kanamycin**				
Xpert KmS (39)	36	0	3	36/37=97.3%
Xpert KmR (01)	0	0	1	
Xpert KmI (03)	1	0	2	
Xpert not done (30)	5	0	25	

Abbreviations: DST, drug susceptibility testing; FQR, fluoroquinolone resistant; FQI, fluoroquinolone indeterminate; FQS, fluoroquinolone susceptible; HI, isoniazid indeterminate; HR, isoniazid resistant; HS, isoniazid susceptible; KmI, kanamycin indeterminate; KmR, kanamycin resistant; KmS, kanamycin susceptible.

^${\$}$ ^Observed agreement: calculated using Cohen's kappa, not taking into account the category ‘not done’, and considering ‘indeterminate’ on Xpert XDR as not in agreement with either susceptible or resistance on phenotypic DST.

^#^Phenotypic DST uses ofloxacin to show whether resistance is present.

## Discussion

Rifampicin DST coverage among bacteriologically confirmed pulmonary TB cases was 95.6%, and was similar for both new cases (95.7%; 3659/3825) and previously treated (95.5%; 406/425) TB patients. However, second-line DST coverage was only 65.8% (48/73) among those with RR-TB. The high rifampicin DST coverage allows the Rwandan NTP to use routinely performed DST on sputum samples for continuous surveillance of pulmonary DR-TB and, as such, estimate the prevalence of RR-TB. WHO guidelines recommend at least 80% rifampicin DST coverage among pulmonary bacteriologically confirmed TB cases.^1^ Experiences from other settings show that it is not easy to achieve this target, either because not all clinics participated,^3^ or because the DST coverage was too low in the participating clinics.^4,5^ Usually DST coverage is better in previously treated patients than in new patients, because previously treated cases are more at risk of RR-TB.^1,2^

With 95.6% coverage, the Rwandan NTP is performing very well. With close to exhaustive sampling, the obtained estimates for RR-TB prevalence could reflect the reality in this setting. This information is key to the NTP, as rifampicin is the core drug of first-line treatment regimens, highly active in patients with rifampicin-susceptible TB.^10,11^ Despite such high DST coverage, the total number of patients notified with RR-TB is far lower than WHO estimates. In 2019, while the WHO estimated 190 RR-TB cases, the NTP notified only 101 (53%) RR-TB cases.^12^ Considering the very high coverage of rifampicin DST among patients with bacteriologically confirmed pulmonary TB among both new and previously treated cases, we believe our data provide a more realistic estimate of the burden of RR-TB among those diagnosed with TB than the estimate provided by the WHO.

The coverage of second-line DST was relatively low. This may have resulted in a poor estimate of the true prevalence of resistance to fluoroquinolone and other second-line drugs among patients with RR-TB. Fluoroquinolone is used as a core drug in RR-TB treatment regimens, hence the importance of estimating the prevalence of resistance to fluoroquinolone among those with RR-TB.^13^ The roll-out of the Xpert XDR cartridge (with DST for fluoroquinolone, second-line injectables, ethionamide and isoniazid) is a real opportunity to improve second-line DST coverage.^14^ Xpert XDR can be performed on the same platform used for rifampicin DST, and will thus be more accessible than phenotypic DST or second-line LPA, which require sophisticated infrastructure, strict infection control measure and highly skilled staff. Moreover, the shorter turnaround time of Xpert facilitates the use of DST results for clinical decision-making.

The prevalence of RR-TB was 1.4% (95% CI 1.02 to 1.78%) and 4.9% (2.80 to 7.00%) among new and previously treated TB cases, respectively. This was at 1.5% (0.76 to 2.24%) and 10.7% (4.09 to 17.31%) in 2015.^7^ Results show a significant reduction of RR-TB among previously treated TB cases (p=0.04). As shown in our previous study, the countrywide roll-out of Xpert MTB/RIF testing over a period of 10 y (2006–2016) reduced the median diagnostic delay (delay between sampling and having the diagnosis available for the clinician) from close to 100 to 1 d, while better access to RR-TB treatment reduced the median therapeutic delay (delay between diagnosis and treatment start) from >50 to 3 d.^9^ Both new cases and previously treated patients diagnosed with TB on smear microscopy were eligible for Xpert testing. Moreover, Xpert MTB/RIF testing is increasingly used as the first test, in both risk groups and also in new cases in Kigali City (Table 1). Better access to Xpert MTB/RIF testing in new cases avoids the use of ineffective rifampicin-based treatment in new cases with initially RR-TB. Without baseline rapid RMP DST, such patients with initially RR-TB would have been treated with a rifampicin-based first-line treatment regimen, causing treatment failure or relapse in about 50%.^11^ Moreover, early highly effective treatment of active RR-TB is the most important intervention to stop RR-TB transmission.^15^ In our setting, both standardized long- and short-treatment regimens (used since July 2014) resulted in >80% success.^9^

The comparison between results from the 2015 DRS and findings from our routine surveillance should be considered with caution. The 2015 DRS used different sampling methods and different tests. In 2015, all sputum smear-positive patients were recruited for the DRS and samples were actively collected and transported the same day to the NRL for testing during the 6-mo study implementation period. LPA and phenotypic DST for both first- and second-line TB drugs were performed on all culture positive samples.^7^ By contrast, routine surveillance mainly relied on Xpert MTB/RIF, either as an initial TB diagnostic test for those with presumptive TB or as a second test in patients with positive sputum smears to exclude RR-TB.

Also, in Bangladesh, continuous surveillance was evaluated. Here, the RR-TB prevalence estimate obtained from DRS was in line with what was found during continuous surveillance. Moreover, changes in RR-TB prevalence were first seen during continuous monitoring, a few years before being confirmed by results from the periodic DRS.^16^ Similarly, the Rwandan NTP could use the data from continuous surveillance to inform clinical and program decision-making and thus better control the RR-TB epidemic.

In Rwanda, TB patients who were close contacts (mainly household contacts) of a known RR-TB case had an 11 times increased risk of being diagnosed with RR-TB, which is in line with the literature.^17^ Close contacts of a RR-TB patient are at risk of primary rifampicin resistance. Kendall et al., in their findings from a transmission modeling analysis of programmatic data, estimated that RR-TB transmission was the cause of RR-TB in 95.9% (68.0 to 99.6%) of all incident RR-TB and 61.3% (16.5 to 95.2%) in previously treated individuals with MDR-TB disease.^18^ Therefore, the Rwandan NTP should continue to prioritize active case finding among contacts of RR-TB cases. Previously treated TB patients had a fourfold increased risk of being diagnosed with RR-TB in Rwanda. RR may be acquired during first-line TB treatment, which is more frequent when the resistance-preventing activity of the treatment regimen is inadequate, particularly in the case of initial isoniazid resistance.^11^ However, recent findings showed that the main cause of RR-TB in previously treated patients is not the development of new resistance but primary resistance, thus already present when the patient was treated a first time for TB.^11^ Living in Kigali doubled the risk of being diagnosed with RR-TB. Kigali City can be considered as a high-risk zone for RR-TB disease, probably due to poverty and crowding in some parts of the city. Therefore, the Rwandan NTP recommends using Xpert as a first test for all those with presumptive TB.^19^

A strength of the present study was the very high coverage of rifampicin DST among all bacteriologically confirmed pulmonary TB patients. Our findings show the reality of the Rwandan NTP with regard to the prevalence of RR. Our experience, which shows that routine surveillance can achieve high coverage for both new and previously treated patients, may inform other high TB burden countries. However, to achieve a higher coverage as in Rwanda, local factors may need to be taken into account. To achieve a high rifampicin DST coverage, DR-TB has to be recognized as a public health priority and staff need to be motivated to meet clearly defined targets. Moreover, in Rwanda, distances between health facilities are relatively short. The greater the distances, the more challenging it is to implement a performing sample collection system.

Our study also had limitations. As recommended in the WHO guidelines on surveillance of drug resistance, our study targeted patients with bacteriologically confirmed pulmonary TB.^1^ We therefore do not show an estimate of the burden of DR-TB in patients with extrapulmonary TB. The main rifampicin DST method was the Xpert MTB/Rif assay. This assay detects *Mycobacterium tuberculosis* complex, and does not show the species detected, limiting the possibility of using a One Health approach during the investigation of drivers of antibiotic resistance.^20^ Moreover, by using the Xpert MTB/RIF assay in a low DR-TB prevalence setting such as Rwanda, a substantial proportion of patients may have been falsely diagnosed with RR-TB, especially when the bacillary load was low.^19^ The RR-TB prevalence may therefore have been overestimated. Indeed, among 70 patients with at least one additional rifampicin DST result at the NRL, RR-TB was confirmed in only 41 patients. Our previously published data showed 50% false RR among new cases and 33% false RR among retreatment patients.^19^ On the other hand, Xpert may also miss resistance, for instance, when both wild-type and resistant populations are present (i.e. heteroresistance), or when the mutation is systematically missed by Xpert.^21^ We plan to systematically correct for false RR in our next round of RR surveillance (planned for the fiscal year July 2022–June 2023). Instead of Xpert MTB/RIF, Xpert Ultra will have been used for rifampicin DST. Xpert XDR in combination with phenotypic DST will be used in all patients diagnosed with RR-TB, which will allow us to reach a better estimate of the prevalence of resistance to second-line drugs among those with RR-TB, and also allow us to further investigate those with RR-TB on Xpert Ultra but rifampicin-susceptible TB on phenotypic DST. In addition, in Kigali City we will estimate the proportion of RR missed on Xpert Ultra by using phenotypic DST among all those with bacteriologically confirmed pulmonary TB. As such we expect to come to an estimate of the RR prevalence corrected for both false and missed RR.

## Conclusions

High DST coverage and routine reporting allowed Rwanda to implement a TB case-based surveillance system for the estimation of RR-TB prevalence in both new and previously treated patients. Improvements are needed to exclude false positive Xpert results and also correct for missed RR to further improve the estimate of RR prevalence in our setting. Moreover, the coverage of second-line DST among those with RR-TB needs to increase to obtain an estimate of the prevalence of resistance to second-line drugs among patients with RR-TB. Once such improvements are in place, the Rwandan NTP will be able to regularly estimate the national DR-TB burden and make decisions based on data reflecting the true treatment needs of patients.

## Data Availability

The data underlying this article will be shared upon reasonable request to the corresponding author.
